# A Geographically Weighted Cost-effectiveness Analysis of Newborn Cytomegalovirus Screening

**DOI:** 10.1093/ofid/ofae311

**Published:** 2024-06-07

**Authors:** Paul M Lantos, Soren Gantt, Mark Janko, Francois Dionne, Sallie R Permar, Karen Fowler

**Affiliations:** Divisions of Pediatric Infectious Diseases, General Internal Medicine, and Occupational and Environmental Medicine, School of Medicine, Duke University, Durham, North Carolina, USA; Duke Global Health Institute, Durham, North Carolina, USA; Departments of Microbiology, Infectious Diseases & Immunology and Pediatrics, Université de Montréal, Montreal, Quebec, Canada; Duke Global Health Institute, Durham, North Carolina, USA; Centre for Clinical Epidemiology and Evaluation, Vancouver, British Columbia, Canada; Department of Pediatrics, Weill Cornell Medicine, New York, New York, USA; Department of Pediatrics, University of Alabama, Birmingham, Alabama, USA

**Keywords:** congenital CMV, cost-effectiveness, geographic information systems, hearing loss, neonatology, screening

## Abstract

**Background:**

Early identification of newborns with congenital cytomegalovirus (CMV) is necessary to provide antiviral therapy and other interventions that can improve outcomes. Prior research demonstrates that universal newborn CMV screening would be the most cost-effective approach to identifying newborns who are infected. CMV is not uniformly prevalent, and it is uncertain whether universal screening would remain cost-effective in lower-prevalence neighborhoods. Our aim was to identify geographic heterogeneity in the cost-effectiveness of universal newborn CMV screening by combining a geospatial analysis with a preexisting cost-effectiveness analysis.

**Methods:**

This study used the CMV testing results and zip code location data of 96 785 newborns in 7 metropolitan areas who had been tested for CMV as part of the CMV and Hearing Multicenter Screening study. A hierarchical bayesian generalized additive model was constructed to evaluate geographic variability in the odds of CMV. The zip code–level odds of CMV were then used to weight the results of a previously published model evaluating universal CMV screening vs symptom-targeted screening.

**Results:**

The odds of CMV were heterogeneous over large geographic scales, with the highest odds in the southeastern United States. Universal screening was more cost-effective and afforded more averted cases of severe hearing loss than targeted testing. Universal screening remained the most cost-effective option even in areas with the lowest CMV prevalence.

**Conclusions:**

Universal newborn CMV screening is cost-effective regardless of underlying CMV prevalence and is the preferred strategy to reduce morbidity from congenital CMV.

## Teaser

A geographically weighted cost-effectiveness analysis demonstrates that universal newborn cytomegalovirus screening remains the most cost-effective strategy to identify newborn cytomegalovirus regardless of local prevalence.

Cytomegalovirus (CMV) is the most common congenital infection and the most common treatable cause of pediatric neurologic disabilities. Approximately 0.5% of live-born infants in the United States are infected with CMV in utero, at least 15% of whom will develop 1 or more neurodevelopmental problems, including sensorineural hearing loss, vestibular dysfunction, visual impairment, intellectual disability, and microcephaly [1, 2]. Early identification of congenital CMV allows not only early antiviral therapy for symptomatic newborns, which improves long-term hearing outcomes, but also early intervention and close audiologic follow-up for all children who are infected to ensure optimal speech and language outcomes [3]. Importantly, however, because the majority of congenital CMV cases are asymptomatic or have subclinical or nonspecific signs at birth, few newborns receive a diagnosis of or directed care for congenital CMV in the absence of newborn CMV screening [4].

There remains no consensus on how best to identify infants with congenital CMV. At present, Minnesota is the only US state with a universal CMV screening program. Several additional states now mandate CMV testing for infants who fail a newborn hearing screen, a strategy that leads to prompt antiviral treatment and the potential for clinical and cost savings [5, 6]. This targeted approach, though, still misses nearly all infants who develop late-onset hearing loss from congenital CMV, including nearly half of those with onset of hearing loss after the newborn period. Previous studies suggest that universal newborn CMV screening would improve hearing and cognitive outcomes among thousands of children, as well as greater total cost savings [7, 8].

Earlier studies typically assumed homogenous rates of congenital CMV disease and care across populations. However, congenital CMV disproportionately affects socially disadvantaged demographic groups, and infections have been shown to be particularly concentrated in communities of color [9–11]. The burden of congenital CMV may therefore be concentrated in disadvantaged communities, and the cost-effectiveness of universal CMV screening may be greater in these communities than in lower-prevalence areas. In this study, we report a geographically weighted cost-effectiveness analysis to quantify the impact of geography on preferential newborn CMV screening strategies.

## METHODS

### Patient Consent Statement

This study was approved by the institutional review boards of the Duke University Health System and the University of Alabama at Birmingham. The requirement for informed consent was waived for this secondary data analysis.

### Design and Data Sources

To identify the scale of geographic variability in the costs and cost savings of different infant CMV testing strategies, we used a geographic model as a weight for a preexisting cost-effectiveness analysis. Our primary data source was the CMV and Hearing Multicenter Screening (CHIMES) study, which investigated universal CMV testing among 100 332 infants at 7 medical centers [12]. Infants were tested by salivary polymerase chain reaction. Only those from zip codes with ≥10 infants (n = 96 903) were retained for this study, of whom a further 118 (0.1%) were excluded because their recorded zip codes could not be reconciled to our national zip code database. The final sample for our analysis comprised 96 785 infants.

Of those enrolled in CHIMES, 4.5 per 1000 tested positive for CMV; 9.6% of these were symptomatic at birth, of whom 7.8% (of all infants enrolled) had sensorineural hearing loss [12]. We obtained data such as residential zip code, race, ethnicity, and the results of CMV testing for infants in the CHIMES database. Race and ethnicity were self-reported by parents or caregivers and categorized as Black, non-Hispanic White, Hispanic White, Asian, American Indian, or multiracial. To evaluate neighborhood-level indicators of deprivation, we obtained a data set of the Social Deprivation Index (SDI) [13]. The SDI is a composite index of 7 socioeconomic variables from the 2015 American Community Survey, tabulated at the zip code level.

Self-reported race is strongly associated with risk and prevalence of CMV, likely reflecting disparities in exposure and the effects of socioeconomic deprivation [9–11, 14]. Because populations within racial and ethnic categories are not evenly distributed geographically, we chose to adjust for race and ethnicity when modeling the spatial distribution of CMV.

We used our previously published cost-effectiveness analysis of CHIMES for this study, in which comprehensive methodological details can be found [8]. Models were originally constructed to evaluate universal screening and targeted testing and incorporated the following assumptions: 1.5% of infants fail hearing screening, 10% of whom will have hearing loss subsequently confirmed; 13.3% of infants with hearing loss have congenital CMV infection; and 25% of infants with congenital CMV would be diagnosed clinically in the absence of screening [2, 7, 15–22]. Additional model assumptions included the costs of CMV testing, follow-up, antiviral administration, follow-up care, and the long-term costs associated with the expected clinical outcomes. Model output, which we used for this study, included (1) the percentage reduction in severe to profound hearing loss enabled by screening and (2) the net costs/savings per newborn, including loss-of-productivity costs, which in 2016 dollars were $21.34 and $10.66 for universal and targeted strategies, respectively [8]. For this study, all costs were converted from 2016 to 2022 dollars by multiplying by 1.22.

### Spatial Models

Our spatial modeling had 2 goals. First, we intended to evaluate covariates associated with the geographic distribution of congenital CMV; second, we intended to generate the predicted prevalence of congenital CMV in each geographic unit (zip code) in our study area. To accomplish this, we fit a hierarchical bayesian spatial model using the statistical programming language R (www.r-project.org) and the statistical package *INLA* [23]. The *INLA* package facilitates construction of hierarchical bayesian models and makes use of integrated nested LaPlace approximation to estimate the posterior distribution. We used default priors for all parameters and hyperparameters.

The outcome variable for our model was the result of CMV testing, dichotomized as positive or negative. Linear covariates included in our model were maternal age, infant race, infant sex, and SDI. Numerical variables were centered on zero by subtracting the mean and were standardized by dividing by the SD. The reference levels for categorical variables were self-reported White race and female sex.

Each infant fell within a zip code and within a medical center, and these identifiers were retained in the model as geographic identifiers. We added a random intercept for medical center and for zip code to account unmeasured variance at these 2 organizational levels. Geostatistical models may violate typical regression assumptions about the independence of observations. This is a result of spatial autocorrelation: the tendency of observations in close spatial proximity to share more unmeasured attributes than observations that are more spatially distant. In turn, this may create nonindependence of observations and spatially correlated error. To address this, our models were conditional autoregressive models based on a Besag spatial correlation structure, which identifies spatially correlated random effects [24]. Spatial proximity in conditional autoregressive models is defined by an adjacency matrix that defines neighbor relationships among the observations.

### Spatial Cost-effectiveness Analysis

From our model output, we extracted the estimated odds of congenital CMV for each zip code included in our study. The odds were multiplied by the percentage reduction in cases of severe and profound hearing loss and by the net costs/savings per newborn. As such, a zip code with an odds of 1 would not modify the global costs and savings of screening. For this analysis, we used an unadjusted model, which did not include variables such as race/ethnicity and SDI. This was so that our spatial weighting was based on the real-world distribution of congenital CMV cases, rather than one attenuated by spatially heterogeneous predictors.

## RESULTS

Our data set had data from 96 785 infants, 433 (0.4%) of whom tested positive for congenital CMV. The infants resided in 701 unique zip codes in 7 metropolitan areas: Dallas, Texas; Jackson, Mississippi; Birmingham, Alabama; Charlotte, North Carolina; Cincinnati, Ohio; Pittsburgh, Pennsylvania; and New Brunswick, New Jersey. The number of infants per study site ranged from 5910 in Jackson to 22 283 in Dallas. Among the 701 zip codes, there were as few as 10 infants and as many as 1345. Mean maternal age was 27.4 years (IQR, 23–32). The racial, ethnic, and sex distribution of the infants is found in Table 1. Hispanic ethnic identity had been combined with race in the source data set.

**Table 1.** Demographics of Infants

	No. (%)
Race/ethnicity	
Asian	4013 (4.1)
Black	23 332 (24.1)
Hispanic	31 794 (32.9)
Multiple	2348 (2.4)
Native American	97 (0.1)
White	35 201 (36.4)
Sex	
Female	47 587 (49.2)
Male	49 198 (50.8)

Fixed variables included infant sex, infant race/ethnicity, maternal age, and zip code–level SDI. Self-reported Black race and multiracial identity were associated with an elevated odds ratio of congenital CMV infection relative to self-reported White race, whereas self-reported Hispanic ethnicity was associated with a lower odds ratio of congenital CMV. Infants born to younger mothers had a higher risk of congenital CMV: for every 6.1-year increase in maternal age, the odds ratio of congenital CMV was 0.51. A higher SDI (more social deprivation) was associated with a slightly higher risk of congenital CMV. Adjustment for these covariates improved model performance over a purely spatial model in which these variables were excluded (Table 2).

**Table 2.** Covariates Associated With Congenital CMV

	Odds Ratio	95% CI^a^
Male sex	1.05	.87–1.27
Race/ethnicity		
Asian	0.43	.14–1.07
Black	**1.57**	**1.17–2.14**
Multiracial	**1.84**	**1.05–3.06**
Native American	3.14	.33–17.18
Hispanic	**0.55**	**.36–.81**
Maternal age	**0.51**	**.45–.58**
Social Deprivation Index	**1.13**	**1.01–1.29**

Bold values are those in which the 95% CI does not contain 1, suggesting a 95% probability that the odds ratio of congenital CMV is not equal to 1. This model incorporated geographic adjacency and random effects for levels of spatial clustering (geographic adjacency, zip code, and metropolitan study site) in addition to these fixed effects.

Abbreviations: 95% CI, credible interval; CMV, cytomegalovirus.

^a^Bayesian 95% CIs.

**Figure 1.**
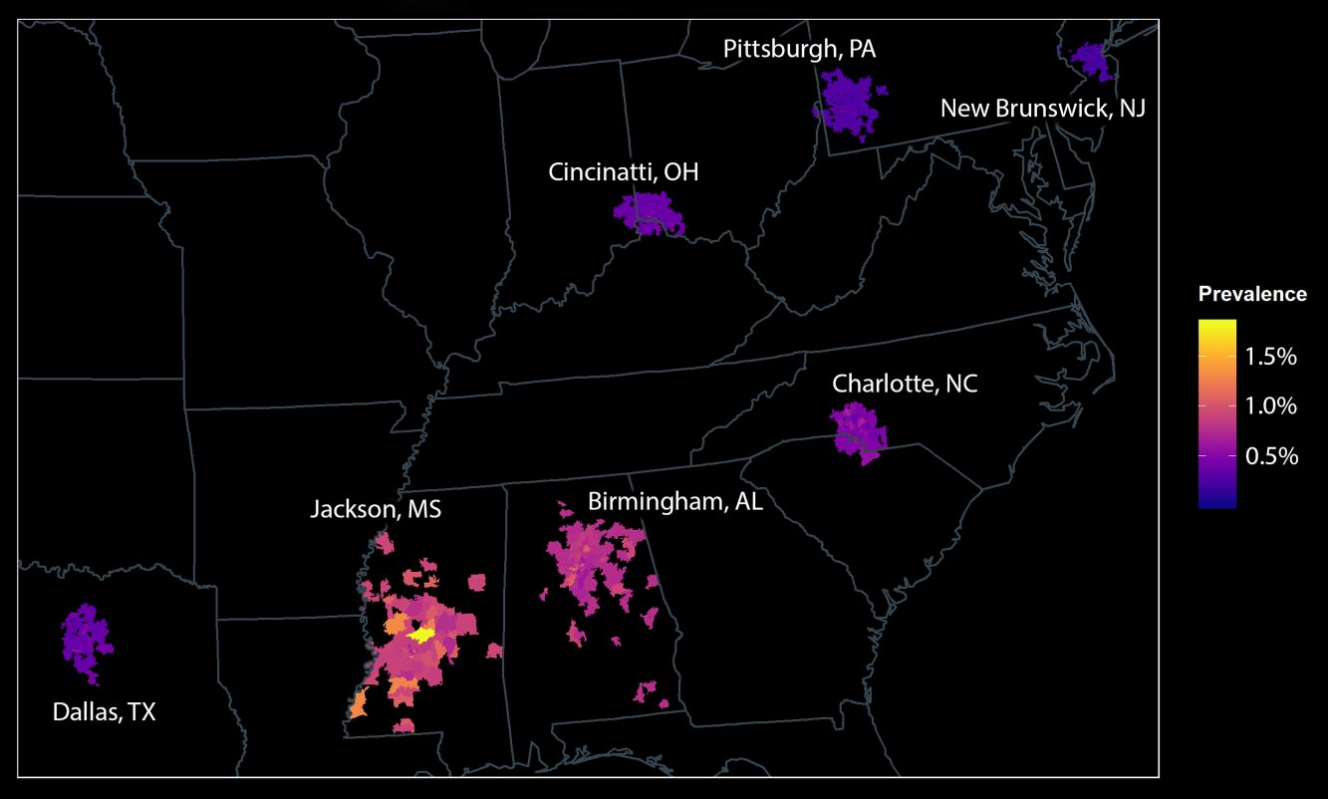
Spatial distribution of congenital CMV among 96 785 infants. Infants were distributed in 701 zip codes in 7 metropolitan areas. The prevalence of congenital CMV was greatest in southeastern sites and was distributed heterogeneously within metropolitan areas. CMV, cytomegalovirus.

We observed geographic variability in the predicted prevalence of congenital CMV at a large scale (among regions of the country) and a small scale (among zip codes in single metropolitan areas; Figure 1). The prevalence ranged from 0.2% to 2% among the 701 zip codes. The prevalence generally increased along a north-to-south gradient, with the highest prevalence found in Jackson and Birmingham. Some geographic heterogeneity was observed within metropolitan areas as well.

**Figure 2.**
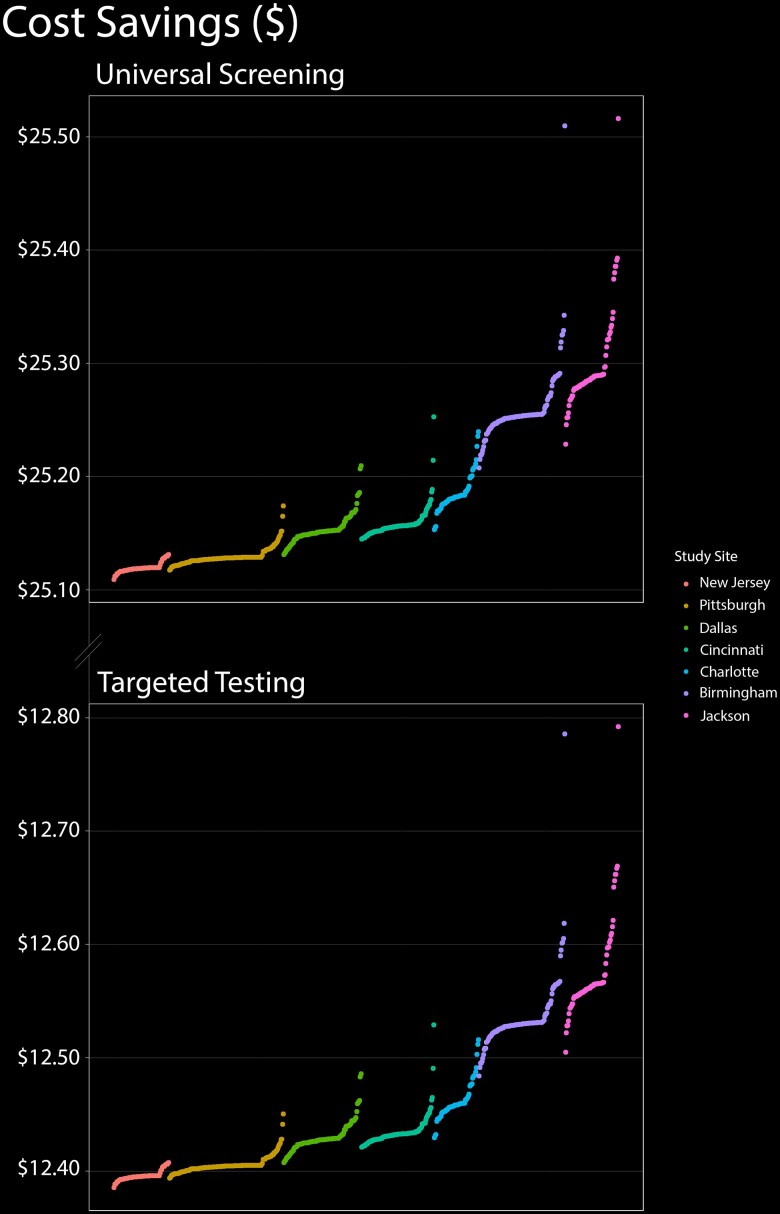
Cost-effectiveness of universal CMV screening and targeted testing as a function of geography. In this figure, the 701 zip codes are distributed sequentially along the x-axis from lowest to highest prevalence within each of the 7 metropolitan areas in our study. Cost-effectiveness is displayed on the y-axis. Regardless of prevalence, universal infant CMV screening was more cost-effective than targeted testing. CMV, cytomegalovirus.

Universal newborn CMV screening resulted in cost savings and reduced morbidity as compared with targeted testing, and the magnitude of this benefit was far greater than any variability in prevalence (Figure 2). Universal screening would be expected to reduce severe cases of congenital CMV-related hearing loss by 7.45% to 7.60%, as opposed to 4.19% to 4.26% for targeted testing. The savings per newborn from universal screening ranged from $25.11 to $25.52, as compared with $12.54 to $12.75 for targeted testing.

## DISCUSSION

We have shown that universal newborn CMV screening for congenital CMV is more cost-effective and clinically effective than targeted testing, even in geographic regions with a lower prevalence of CMV. This supports universal newborn CMV screening as a national strategy to reduce the costs and morbidity from CMV. While universal screening in high-prevalence communities confers the greatest value, it is clearly the preferred strategy regardless of prevalence.

Numerous studies have reported markedly higher CMV seroprevalence among racial and ethnic minorities than among Whites [10, 11, 15, 25–29]. As compared with individuals who identify as White, there are greater infection and mortality rates from congenital CMV among infants born to families that identify as Black [12, 30]. CMV seroprevalence is greater in neighborhoods with larger Black populations, and this geographic distribution is associated with measures of social deprivation [9]. Congenital CMV and other CMV-related diseases must be seen as one of the major neglected infections of poverty in the United States [31, 32]. Additionally, infant hearing loss appears to be more prevalent in disadvantaged neighborhoods, particularly those with predominantly minority populations, and this disparity may be related to the prevalence of congenital CMV in these communities [14].

There remains no uniform public health strategy for identifying CMV-infected infants in the United States. In most states, infant CMV testing is performed ad hoc—that is, when there is clinical suspicion of congenital CMV by a treating clinician. Some states have either passed or are considering legislation for testing all infants who fail hearing screening. This systematic symptom-targeted strategy clearly offers clinical and financial advantages over ad hoc testing [8]. Targeted testing, however, has the disadvantage of missing asymptomatic infants who will develop late hearing loss, as well as those with nonauditory manifestations of congenital CMV for whom antiviral therapy could improve clinical outcomes. Universal screening would be the most cost-effective strategy and would yield the most widespread clinical benefit, regardless of geography. Nevertheless, both strategies were associated with greater relative benefits in more disadvantaged communities.

Our study is a novel combination of decision analysis and geospatial modeling, in which we have evaluated the geographic impact of a complex public health decision. Our study has certain limitations that should be discussed. Source data were aggregated by zip code, and zip codes do not necessarily correspond to neighborhood boundaries or even municipal boundaries in some cases. Our demographic analysis relied on self-reported race and ethnicity, which are to a large degree social constructs that do not necessarily correspond to genetic or geographic ancestry; moreover, the race and ethnicity choices available to our study participants may not have fully captured their racial or ethnic identities. Our analytic strategy of weighting cost-effectiveness by prevalence does not necessarily account for unmeasured geographic differences in medical costs, access to medical care, and local trends in insurance. As such, there may remain unmeasured geographic factors that influence local variability in cost-effectiveness. The underlying cost models are limited by the accuracy of their inputs, particularly the cost per test and costs related to clinical care. The start-up costs for a screening program were not incorporated in the initial model. Additionally, as interventions and long-term outcomes occur over time, the model may be sensitive to the chosen discount rate.

Universal application of a public health intervention must take into account costs, savings, and clinical and public health benefits applied over a range of risk levels, as higher-risk subpopulations may stand to benefit more than others. Our data indicate that newborn CMV screening may be of particular value in communities with the highest burden of congenital CMV, which are also the most socioeconomically disadvantaged. As such, newborn CMV screening has important health equity implications. Importantly, while morbidity from congenital CMV may be concentrated demographically and geographically, the advantages of universal screening over targeted testing would be realized even in lower-risk settings. Thus, our goal should be universal newborn CMV testing strategies so that the greatest number of infected infants can benefit.

## CONCLUSIONS

Our study provides additional support for universal newborn screening for CMV. Not only does this strategy appear to be the most cost-effective and clinically effective overall, but its benefits remain even in areas with lower CMV prevalence. Future research should prospectively evaluate the costs and benefits of universal CMV screening in practice.

## Supplementary Material

ofae311_Supplementary_Data

## Supplementary Data

Supplementary materials are available at *Open Forum Infectious Diseases* online. Consisting of data provided by the authors to benefit the reader, the posted materials are not copyedited and are the sole responsibility of the authors, so questions or comments should be addressed to the corresponding author.

## Notes

***Author contributions.*** P. M. L. conceptualized and designed the study, conducted the analyses, and drafted and refined the manuscript. S. G., M. J., F. D., S. R. P., and K. F. designed and refined the study, interpreted the findings, and refined the manuscript. All authors approved the final manuscript as submitted and agree to be accountable for all aspects of the work.

***Financial support.*** This work was supported by the National CMV Foundation (to P. M. L.).
