# Supplementary material for: A Geographically Weighted Cost-effectiveness Analysis of Newborn Cytomegalovirus Screening
Source: Open Forum Infect Dis. 2024 Jun 7;11(6):ofae311. doi: 10.1093/ofid/ofae311 (PMC11200186; doi:10.1093/ofid/ofae311)
Supplement: ofae311_Supplementary_Data [file ofae311_supplementary_data.pdf]

CHIMES INLA Models

```
## Loading required package: sp

## Loading required package: Matrix

## This is INLA_17.12.15 built 2017-12-15 13:36:45 UTC.
## See www.r-inla.org/contact-us for how to get help.

## Warning: package 'sf' was built under R version 3.6.1

## Linking to GEOS 3.6.1, GDAL 2.2.3, PROJ 4.9.3

## Loading required package: spData

## To access larger datasets in this package, install the spDataLarge
## package with: `install.packages('spDataLarge',
## repos='https://nowosad.github.io/drat/', type='source')`

## -- Attaching packages --------------------------------------------------------------------------------

## v ggplot2 3.2.0     v purrr   0.3.2
## v tibble  2.1.3     v dplyr   0.8.3
## v tidyr   0.8.3     v stringr 1.4.0
## v readr   1.3.1     v forcats 0.4.0

## -- Conflicts ---------------------------------------------------------------------------------------- t
## x tidyr::expand() masks Matrix::expand()
## x dplyr::filter() masks stats::filter()
## x dplyr::lag()    masks stats::lag()

## Loading required package: viridisLite
```

Zip code polygons

```
ggplot() +
  geom_sf(data=zipcodes, fill="lightblue" , color="darkblue") +
  geom_sf(data=st, fill="NA", color="gray" ) +
  coord_sf(xlim = c( -90000 , 1820000 ) , ylim= c( 880000 , 2150000 ) ) +
  theme_bw()
```

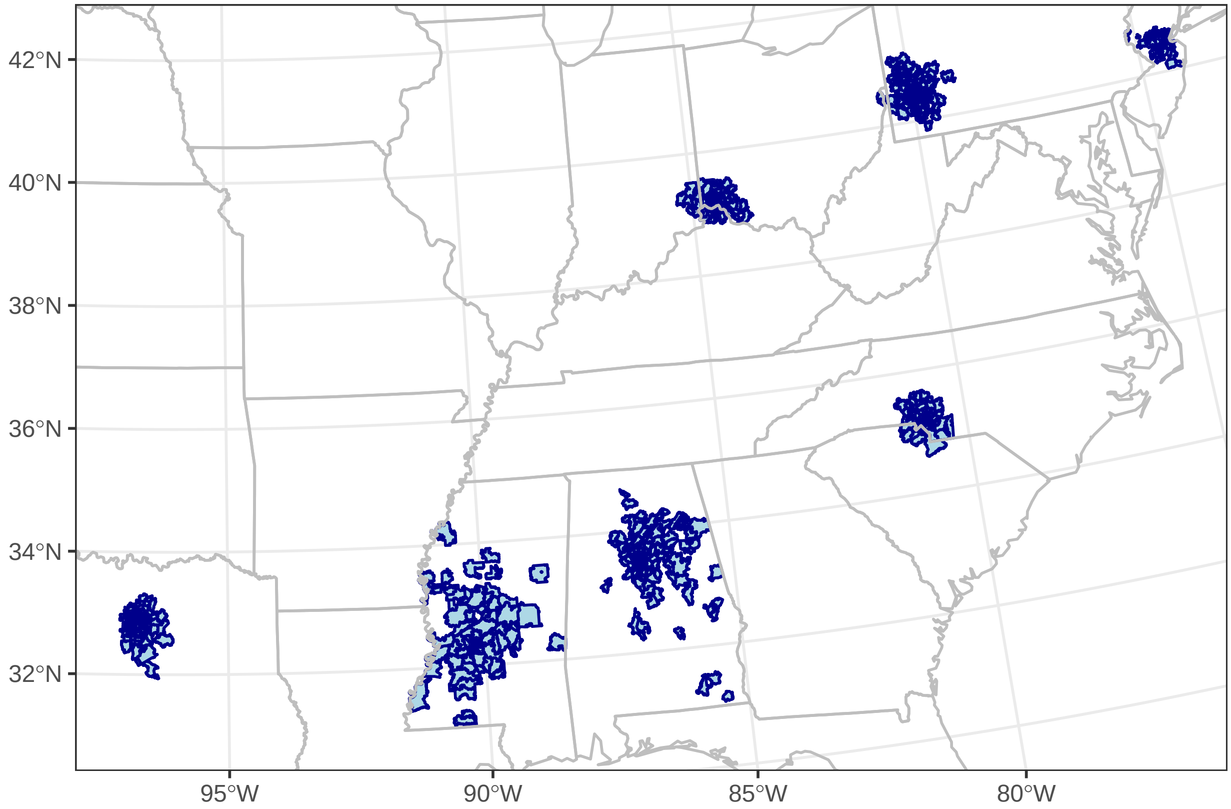

Create adjacency matrix

1. Obtain centroids; define 6 nearest neighbors
2. Create matrix from neighbors

```
centroids_sf <- st_centroid( st_geometry( zipcodes ), of_largest_polygon=TRUE)
coords_sf <- st_coordinates( centroids_sf )
neigh <- knn2nb( knearneigh( coords_sf, k=6 ) )
w1 <- nb2mat(make.sym.nb(neigh), style="B")

plot.nb(neigh, coords_sf, col="#036564", points=FALSE)
```

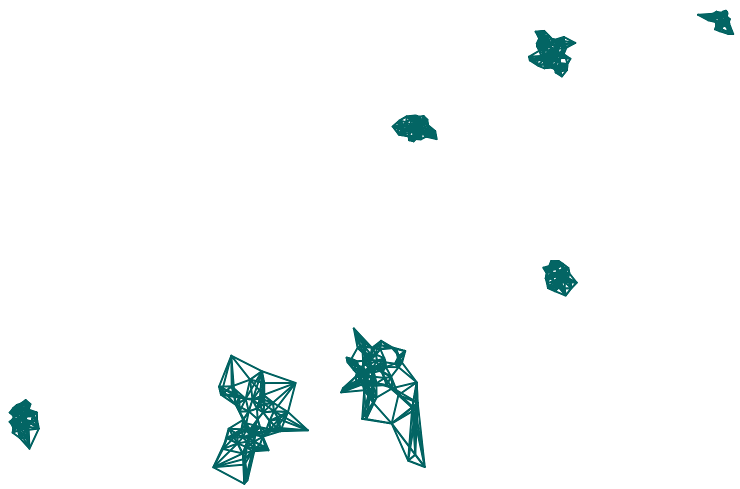

INLA models:

1. Unadjusted

```
formula1 = result ~ # CMV testing result
  f( zipcode , model = "iid" ) + # non-spatial random effects for zipcodes
  f( site , model = "iid" )  # non-spatial random effects for study site

result1 = inla( formula1 , family="binomial" , data=cmvdata ,
                control.predictor = list( compute=T ) ,
                control.compute = list( dic = T ) )
```

2. Unadjusted, including spatial autoregressive term for zipcode

```
formula2 = result ~ # CMV testing result
  f( ID, model = "besag", graph = w1 ) + # spatial random effects for zipcodes
  f( zipcode , model = "iid" ) + # non-spatial random effects for zipcodes
  f( site , model = "iid" )  # non-spatial random effects for study site

result2 = inla( formula2 , family="binomial" , data=cmvdata ,
                control.predictor = list( compute=T ) ,
                control.compute = list( dic = T ) )
```

3. Adjusted for individual variables race, maternal age, and gender

```
formula3 = result ~ race + z_mage + infantsex +
  f( ID, model = "besag", graph = w1 ) +
  f( zipcode , model = "iid" ) +
  f( site , model = "iid" )
```

```
result3 = inla( formula3 , family="binomial" , data=cmvdata ,
                control.predictor = list( compute=T ) ,
                control.compute = list( dic = T ) )
```

4. Adjusted for both individual variables and SDI

```
formula4 = result ~ race + z_mage + infantsex + z_sdi +
  f( ID, model = "besag", graph = w1 ) +
  f( zipcode , model = "iid" ) +
  f( site , model = "iid" )

result4 = inla( formula4 , family="binomial" , data=cmvdata ,
                control.compute=list(dic=T) ,
                control.predictor = list(compute=T))
```

5. Adjusted only for Social Deprivation Index (SDI)

```
formula5 = result ~ z_sdi +
  f( ID, model = "besag", graph = w1 ) +
  f( zipcode , model = "iid" ) +
  f( site , model = "iid" )

result5 = inla( formula5 , family="binomial" , data=cmvdata ,
                control.predictor = list( compute=T ) ,
                control.compute = list( dic = T ) )
```

6. Adjusted for SDI and for percent non-white population

```
formula6 = result ~ z_sdi + pct_nw +
  f( ID, model = "besag", graph = w1 ) +
  f( zipcode , model = "iid" ) +
  f( site , model = "iid" )

result6 = inla( formula6 , family="binomial" , data=cmvdata ,
                control.compute=list(dic=T) ,
                control.predictor = list(compute=T))
```

Spatial distribution of CMV Prevalence

Average mean prevalence by zipcode

```
temp1a <- data.frame( cbind( "zipcode" = result2$.args$data$zipcode ,
                             "unadj" = result2$summary.fitted.values$mean ) )
temp1b <- data.frame ( temp1a %>% group_by( zipcode ) %>%
                         summarize( mean = mean( unadj ) ) )
zipcodes$unadjusted <- temp1b$mean
```

```
range( zipcodes$unadjusted )
```

```
## [1] 0.001992075 0.018160667
```

Plot spatial distribution of unadjusted model estimates:

```
ggplot( ) +
  geom_sf( data=zipcodes , aes( fill=unadjusted ), color="NA" ) +
  scale_fill_viridis( option="D" ,
                      limits= c( 0.001992074 , 0.018160668 ) , "Prevalence"  ) +
  geom_sf( data=st, fill="NA" , color="gray" ) +
```

```
  coord_sf( xlim = c( -90000 , 1820000 ) , ylim= c( 880000 , 2150000 ) ) +
  theme_bw( )
```

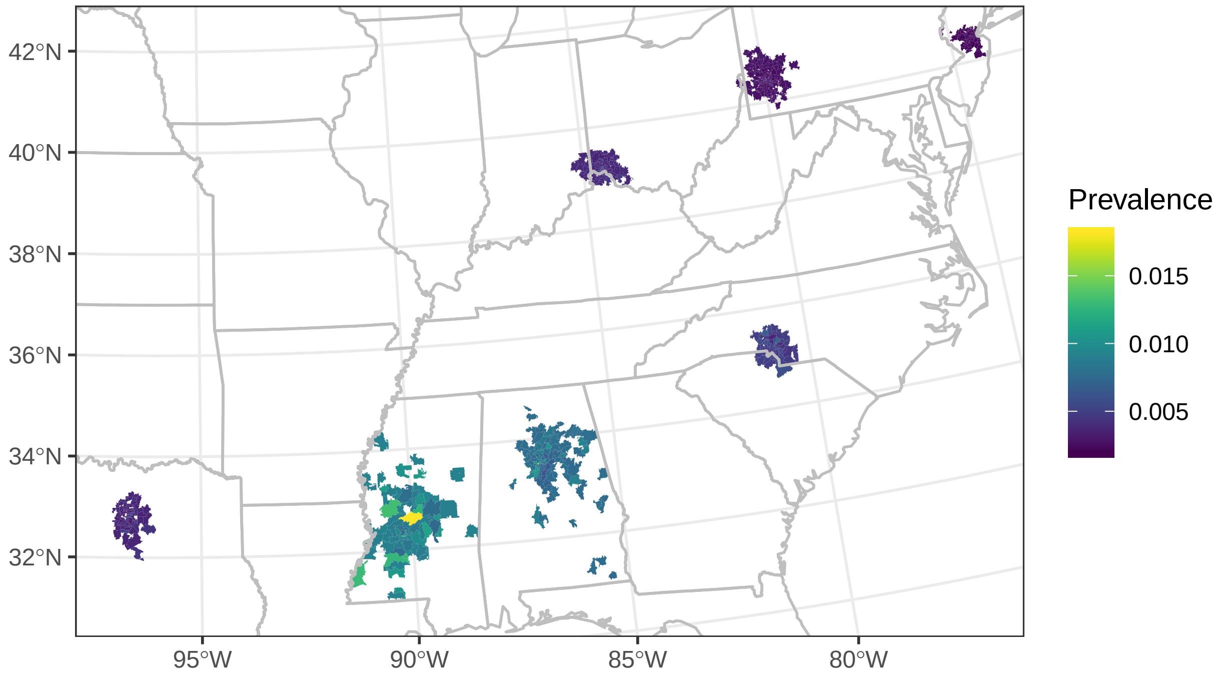


```
ggplot( ) +
  geom_sf( data=zipcodes %>% filter( site=="Jackson" ) ,
           aes( fill=unadj ), color="NA" ) +
  scale_fill_viridis( option="D" ,
                      limits= c( 0.001992074 , 0.018160668 ) , "Prevalence"  ) +
  theme_bw( ) +
  ggtitle( "Jackson: 0.007 to 0.018" )
```

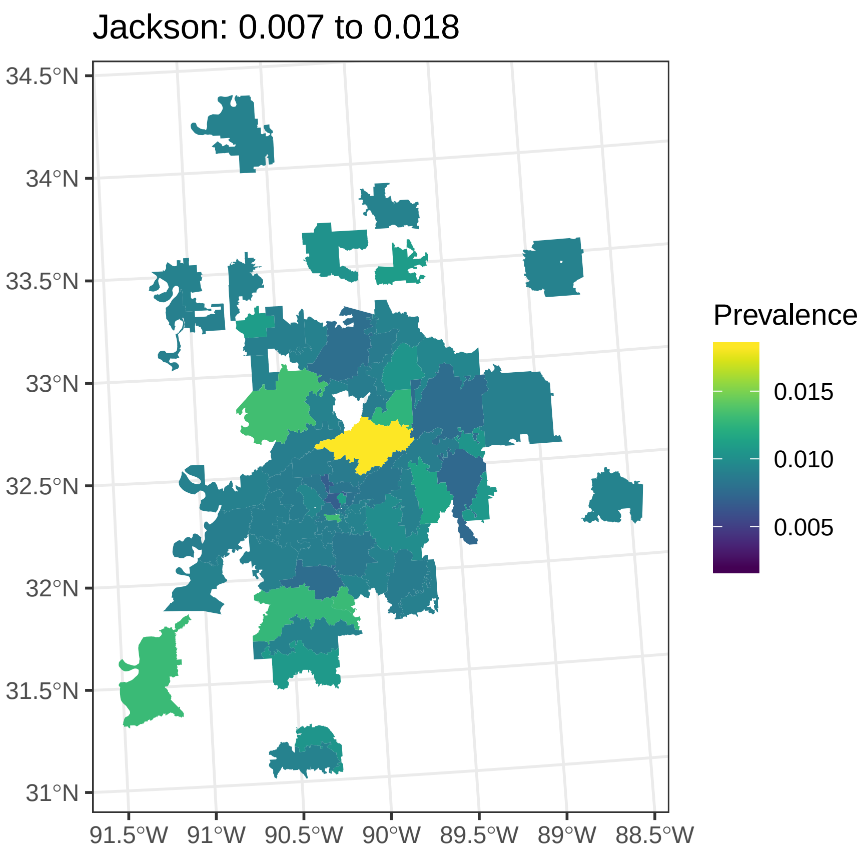


```r
ggplot( ) +
  geom_sf( data=zipcodes %>% filter( site=="Birmingham" ) ,
           aes( fill=unadj ) , color="NA" ) +
  scale_fill_viridis( option="D" ,
                      limits= c( 0.001992074 , 0.018160668 ) , "Prevalence"  ) +
  theme_bw( ) +
  ggtitle( "Birmingham: 0.006 to 0.018" )
```

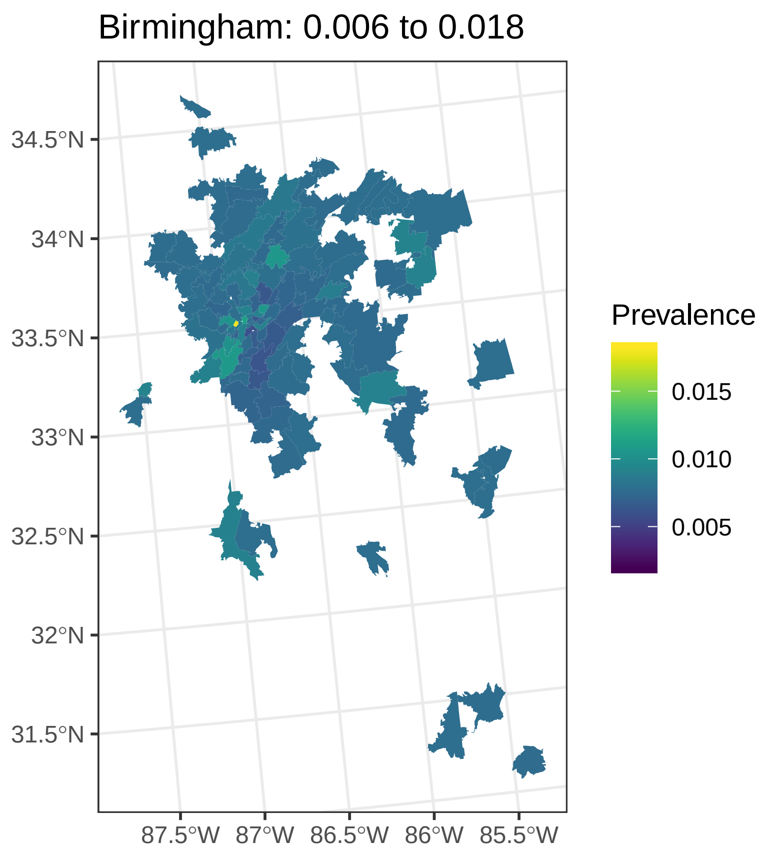


```
ggplot( ) +
  geom_sf( data=zipcodes %>% filter( site=="Dallas" ),
           aes( fill=unadj ), color="NA" ) +
  scale_fill_viridis( option="D" ,
                      limits= c( 0.001992074 , 0.018160668 ) , "Prevalence"  ) +
  theme_bw( ) +
  ggtitle( "Dallas: 0.003 to 0.006" )
```

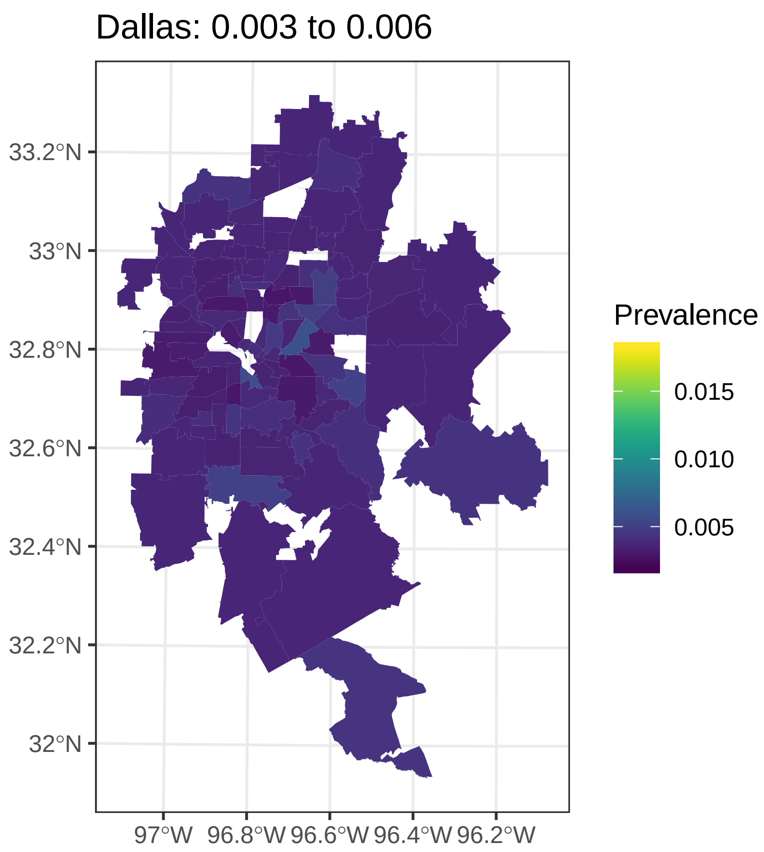


```
ggplot( ) +
  geom_sf( data=zipcodes %>% filter(site=="Charlotte") ,
           aes( fill=unadj ) , color="NA" ) +
  scale_fill_viridis( option="D" ,
                      limits= c( 0.001992074 , 0.018160668 ) , "Prevalence"  ) +
  theme_bw( ) +
  ggtitle( "Charlotte: 0.004 to 0.007" )
```

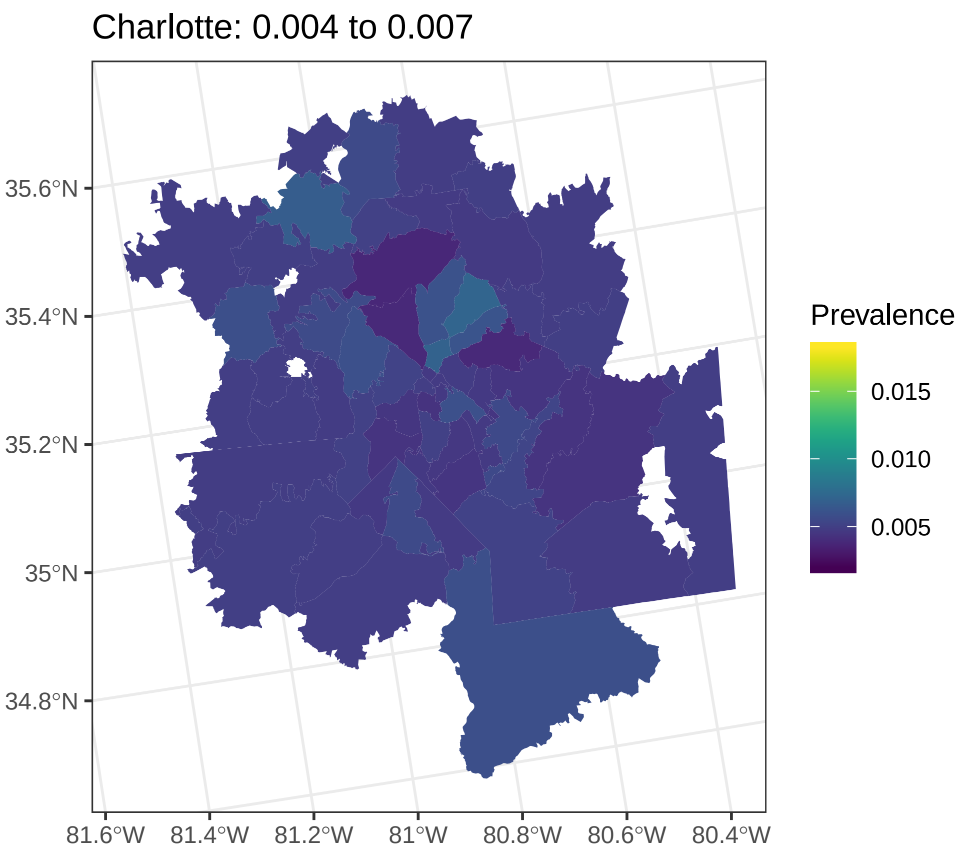


```
ggplot( ) +
  geom_sf( data=zipcodes %>% filter( site=="Cincinatti" ) ,
           aes( fill= unadj ) , color="NA" ) +
  scale_fill_viridis( option="D" ,
                      limits= c( 0.001992074 , 0.018160668 ) , "Prevalence"  ) +
  theme_bw( ) +
  ggtitle( "Cincinatti: 0.003 to 0.008" )
```

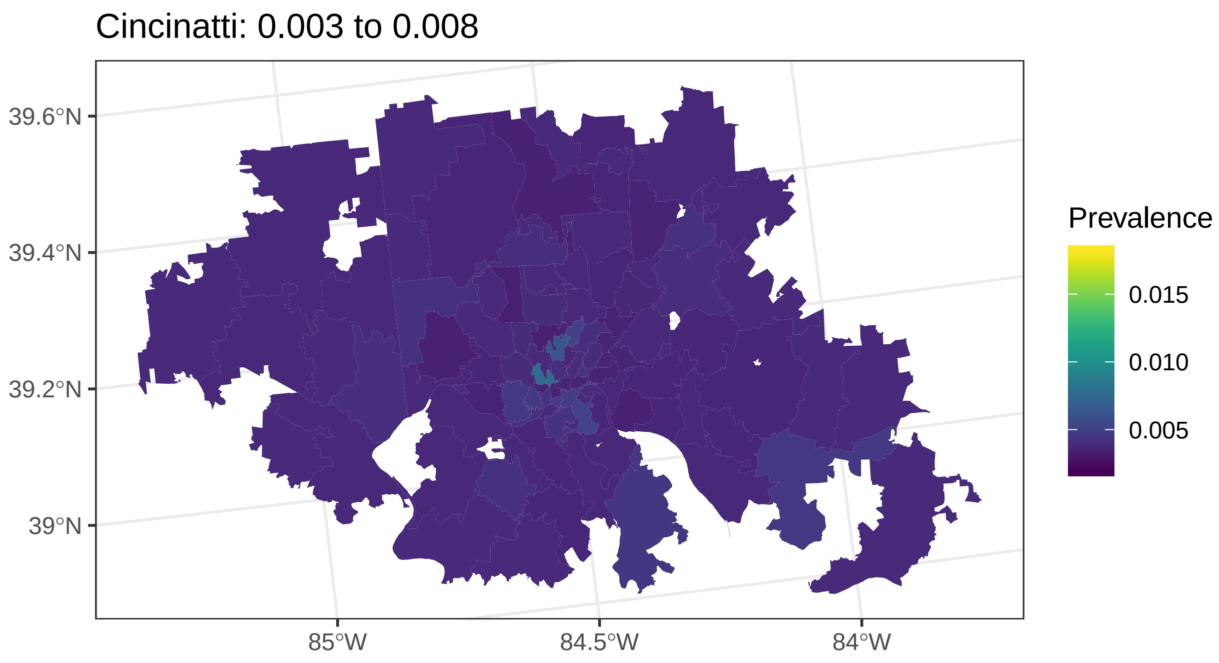


```r
ggplot( ) +
  geom_sf( data=zipcodes %>% filter( site=="Pittsburgh" ) ,
           aes( fill= unadj ) , color="NA" ) +
  scale_fill_viridis( option="D" ,
                      limits= c( 0.001992074 , 0.018160668 ) , "Prevalence"  ) +
  theme_bw( ) +
  ggtitle( "Pittsburgh: 0.002 to 0.005" )
```

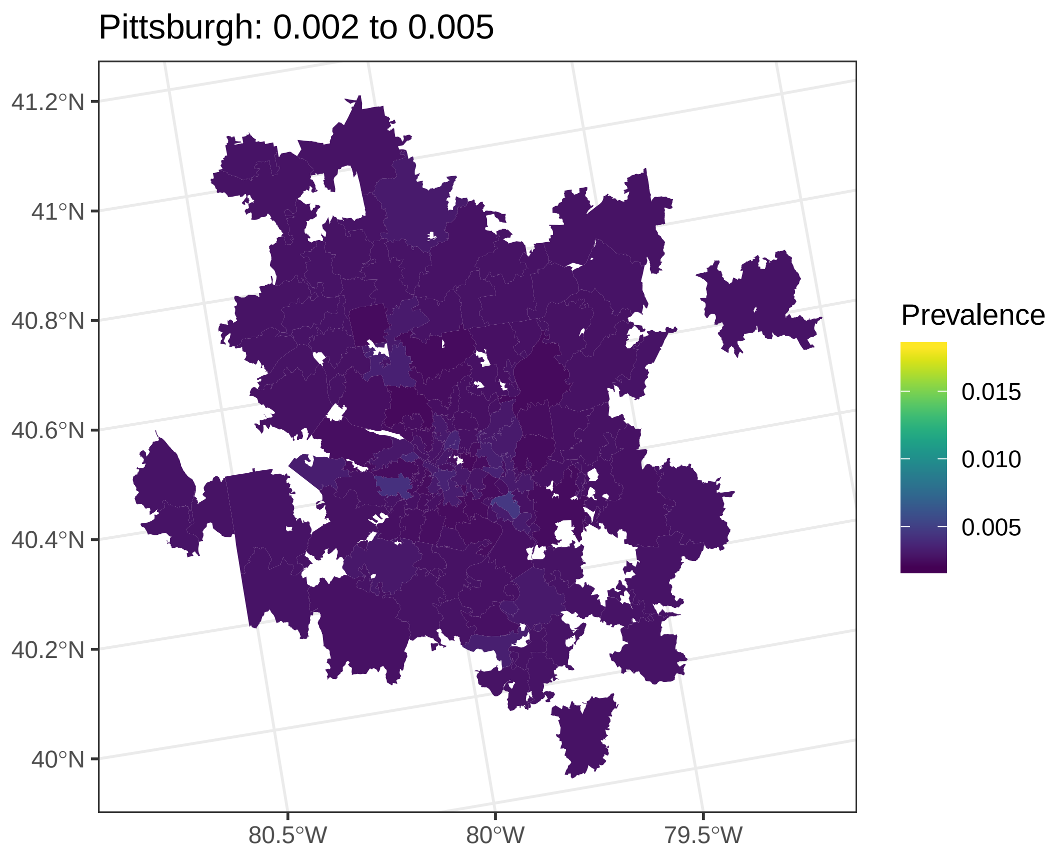


```
ggplot( ) +
  geom_sf( data=zipcodes %>% filter( site=="New Jersey" ) ,
           aes( fill= unadj ) , color="NA" ) +
  scale_fill_viridis( option="D" ,
                      limits= c( 0.001992074 , 0.018160668 ) , "Prevalence"  ) +
  theme_bw( ) +
  ggtitle( "New Jersey: 0.002 to 0.003" )
```

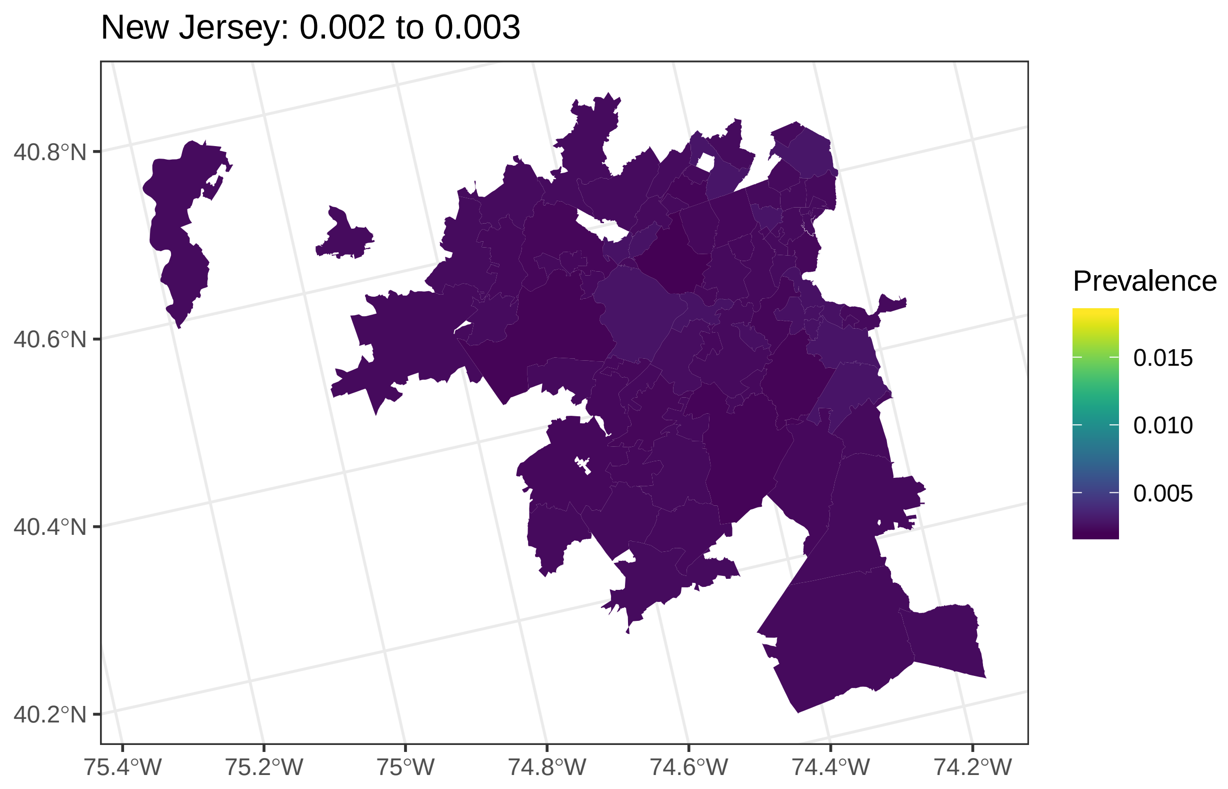

Spatial distribution of CMV Prevalence after adjustment

Average mean prevalence by zipcode

```
temp2a <- data.frame( cbind( "zipcode" = result4$.args$data$zipcode ,
                             "unadj" = result4$summary.fitted.values$mean ) )
temp2b <- data.frame ( temp2a %>% group_by( zipcode ) %>%
                         summarize( mean = mean( unadj ) ) )
zipcodes$adjusted <- temp2b$mean

range( zipcodes$adjusted )
```

```
## [1] 0.0007884698 0.0134486657
```

```
ggplot( ) +
  geom_sf( data=zipcodes , aes( fill=adjusted ), color="NA" ) +
  scale_fill_viridis( option="D" ,
                      limits= c( 0.001992074 , 0.018160668 ) , "Prevalence"  ) +
  geom_sf( data=st, fill="NA" , color="gray" ) +
  coord_sf( xlim = c( -90000 , 1820000 ) , ylim= c( 880000 , 2150000 ) ) +
  theme_bw( )
```

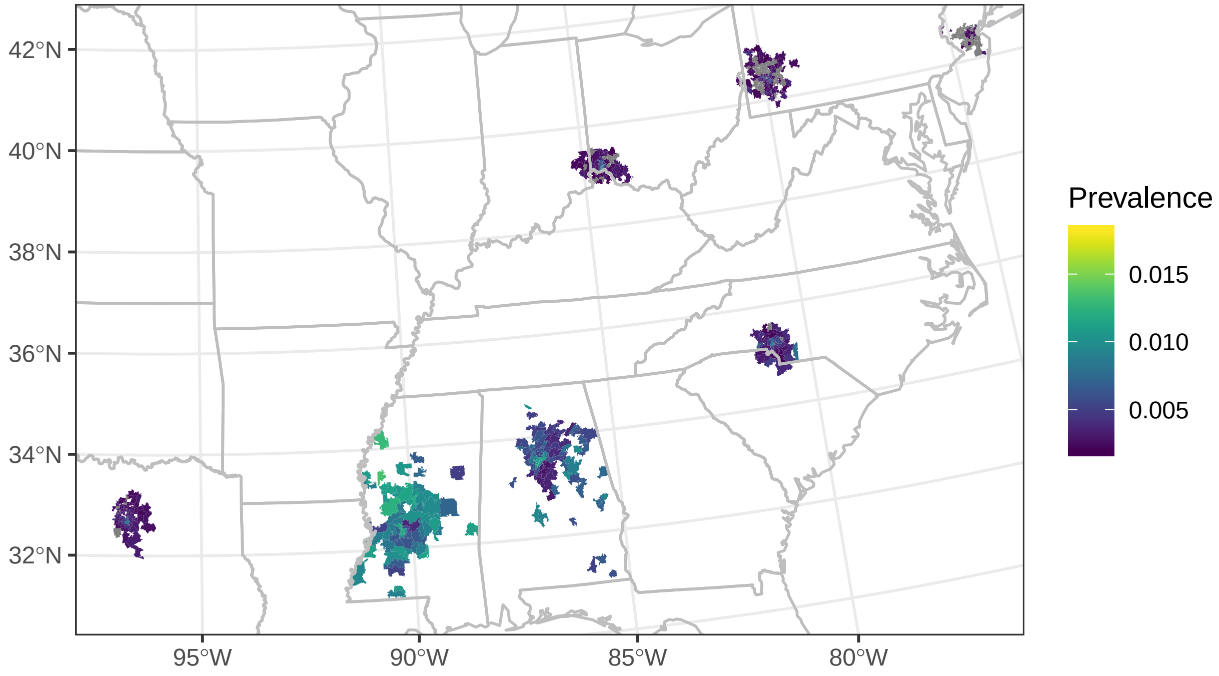

Compare DIC from models

```
##     Model      DIC
## 1 Model 1 5450.839
## 2 Model 2 5440.575
## 3 Model 3 5235.386
## 4 Model 4 5235.526
## 5 Model 5 5418.838
## 6 Model 6 5419.806
```

View model summaries

```
summary( result1 )
```

```
##
## Call:
## c("inla(formula = formula0, family = \"binomial\", data = cmvdata, ",  "    control.compute = list(di
##
## Time used:
##  Pre-processing    Running inla Post-processing           Total
##          4.2744     143028.9822         15.8394     143049.0959
##
## Fixed effects:
##                mean     sd 0.025quant 0.5quant 0.975quant    mode   kld
## (Intercept) -5.4668 0.2115    -5.8928  -5.4658    -5.0469 -5.4646 2e-04
##
## Random effects:
## Name   Model
```

```
##  zipcode   IID model
## site   IID model
##
## Model hyperparameters:
##                         mean    sd 0.025quant 0.5quant 0.975quant  mode
## Precision for zipcode 7.788 4.250      3.042    6.671      18.91 5.109
## Precision for site    5.684 3.057      1.947    4.975      13.51 3.869
##
## Expected number of effective parameters(std dev): 41.40(28.15)
## Number of equivalent replicates : 2337.70
##
## Deviance Information Criterion (DIC) ...............: 5450.84
## Deviance Information Criterion (DIC, saturated) ....: 5450.81
## Effective number of parameters .....................: 44.68
##
## Marginal log-Likelihood:  -2748.43
## Posterior marginals for linear predictor and fitted values computed
```

```r
summary( result2 )
```

```
##
## Call:
## c("inla(formula = formula1, family = \"binomial\", data = cmvdata, ",  "    control.compute = list(di
##
## Time used:
##  Pre-processing    Running inla Post-processing           Total
##          0.6477       9827.2334          5.7815       9833.6626
##
## Fixed effects:
##                mean     sd 0.025quant 0.5quant 0.975quant    mode kld
## (Intercept) -5.4995 0.2051    -5.9157  -5.4981    -5.0916 -5.4953   0
##
## Random effects:
## Name   Model
##  ID   Besags ICAR model
## zipcode   IID model
## site   IID model
##
## Model hyperparameters:
##                             mean        sd 0.025quant  0.5quant 0.975quant
## Precision for ID      18627.318 20776.314   1399.614 12370.795   73486.35
## Precision for zipcode     8.416     4.634      2.996     7.269      20.49
## Precision for site        5.010     3.003      1.337     4.333      12.67
##                            mode
## Precision for ID      3851.441
## Precision for zipcode    5.601
## Precision for site       3.136
##
## Expected number of effective parameters(std dev): 61.35(19.07)
## Number of equivalent replicates : 1577.46
##
## Deviance Information Criterion (DIC) ...............: 5440.57
## Deviance Information Criterion (DIC, saturated) ....: 5440.54
## Effective number of parameters .....................: 61.21
##
```

```
## Marginal log-Likelihood:  -3411.16
## Posterior marginals for linear predictor and fitted values computed
```

```r
summary( result3 )
```

```
##
## Call:
## c("inla(formula = formula2, family = \"binomial\", data = cmvdata, ",  "    control.compute = list(di
##
## Time used:
##  Pre-processing    Running inla Post-processing           Total
##          1.2544      11130.4721          5.6718      11137.3983
##
## Fixed effects:
##                        mean     sd 0.025quant 0.5quant 0.975quant    mode
## (Intercept)         -6.6381 0.5158    -7.7482  -6.6024    -5.7225 -6.5287
## raceBlack            1.4274 0.5164     0.5110   1.3917     2.5396  1.3182
## raceHispanic         0.3356 0.5298    -0.6112   0.3016     1.4704  0.2319
## raceMultiple         1.5454 0.5679     0.5167   1.5144     2.7472  1.4514
## raceNative American  2.0063 1.1241    -0.4607   2.1047     3.9441  2.3128
## raceWhite            0.8564 0.5158    -0.0581   0.8205     1.9678  0.7465
## z_mage              -0.6831 0.0612    -0.8043  -0.6827    -0.5642 -0.6819
## infantsexMale        0.0509 0.0967    -0.1387   0.0508     0.2408  0.0507
##                     kld
## (Intercept)           0
## raceBlack             0
## raceHispanic          0
## raceMultiple          0
## raceNative American   0
## raceWhite             0
## z_mage                0
## infantsexMale         0
##
## Random effects:
## Name   Model
##  ID   Besags ICAR model
## zipcode   IID model
## site   IID model
##
## Model hyperparameters:
##                           mean       sd 0.025quant 0.5quant 0.975quant
## Precision for ID      20867.37 19875.53   1460.859 15094.16   73229.01
## Precision for zipcode 21570.66 20255.92   1457.865 15718.50   74511.64
## Precision for site       33.27    38.97      4.476    21.60     133.39
##                          mode
## Precision for ID      3999.14
## Precision for zipcode 3984.71
## Precision for site      10.72
##
## Expected number of effective parameters(std dev): 12.07(1.021)
## Number of equivalent replicates : 8018.68
##
## Deviance Information Criterion (DIC) ...............: 5235.39
## Deviance Information Criterion (DIC, saturated) ....: 5235.36
## Effective number of parameters .....................: 12.92
```

```
##
## Marginal log-Likelihood:  -3318.85
## Posterior marginals for linear predictor and fitted values computed
```

```r
summary( result4 )
```

```
##
## Call:
## c("inla(formula = formula4, family = \"binomial\", data = cmvdata, ",  "    control.compute = list(di
##
## Time used:
##  Pre-processing    Running inla Post-processing           Total
##          0.9072      10320.3872          7.0000      10328.2945
##
## Fixed effects:
##                        mean     sd 0.025quant 0.5quant 0.975quant    mode
## (Intercept)         -6.6235 0.5125    -7.7278  -6.5875    -5.7154 -6.5130
## raceBlack            1.3025 0.5187     0.3816   1.2669     2.4192  1.1934
## raceHispanic         0.2452 0.5297    -0.7009   0.2111     1.3801  0.1410
## raceMultiple         1.4522 0.5695     0.4201   1.4213     2.6568  1.3585
## raceNative American  1.9852 1.1241    -0.4817   2.0836     3.9229  2.2918
## raceWhite            0.8407 0.5152    -0.0726   0.8047     1.9511  0.7306
## z_mage              -0.6660 0.0617    -0.7882  -0.6656    -0.5461 -0.6648
## infantsexMale        0.0519 0.0967    -0.1376   0.0519     0.2418  0.0517
## z_sdi                0.1303 0.0645     0.0046   0.1299     0.2578  0.1291
##                     kld
## (Intercept)           0
## raceBlack             0
## raceHispanic          0
## raceMultiple          0
## raceNative American   0
## raceWhite             0
## z_mage                0
## infantsexMale         0
## z_sdi                 0
##
## Random effects:
## Name   Model
##  ID   Besags ICAR model
## zipcode   IID model
## site   IID model
##
## Model hyperparameters:
##                            mean       sd 0.025quant 0.5quant 0.975quant
## Precision for ID       18258.40 18218.80   1267.088 12876.38   66455.51
## Precision for zipcode 19412.42 18863.10   1248.396 13854.24   69214.76
## Precision for site       65.90   116.20      5.583    33.42     327.36
##                           mode
## Precision for ID      3494.92
## Precision for zipcode 3365.17
## Precision for site      13.15
##
## Expected number of effective parameters(std dev): 12.57(1.321)
## Number of equivalent replicates : 7702.35
##
```

```
## Deviance Information Criterion (DIC) ...............: 5235.53
## Deviance Information Criterion (DIC, saturated) ....: 5235.50
## Effective number of parameters .....................: 13.78
##
## Marginal log-Likelihood:  -3322.65
## Posterior marginals for linear predictor and fitted values computed
```

```r
summary( result5 )
```

```
##
## Call:
## c("inla(formula = formula3, family = \"binomial\", data = cmvdata, ",  "    control.compute = list(di
##
## Time used:
##  Pre-processing    Running inla Post-processing           Total
##          1.6797      15178.2639          8.3590      15188.3026
##
## Fixed effects:
##                mean     sd 0.025quant 0.5quant 0.975quant    mode kld
## (Intercept) -5.6283 0.1718    -5.9727  -5.6282    -5.2863 -5.6288   0
## z_sdi        0.3560 0.0610     0.2380   0.3554     0.4775  0.3542   0
##
## Random effects:
## Name   Model
##  ID   Besags ICAR model
## zipcode   IID model
## site   IID model
##
## Model hyperparameters:
##                         mean        sd 0.025quant  0.5quant 0.975quant
## Precision for ID      22571.49 19216.911   3439.985 17299.707   73102.99
## Precision for zipcode    14.90    13.777      2.534    10.909      51.22
## Precision for site        9.25     7.218      1.999     7.266      28.25
##                         mode
## Precision for ID      9191.639
## Precision for zipcode    6.137
## Precision for site       4.622
##
## Expected number of effective parameters(std dev): 36.33(18.17)
## Number of equivalent replicates : 2664.24
##
## Deviance Information Criterion (DIC) ...............: 5418.84
## Deviance Information Criterion (DIC, saturated) ....: 5418.81
## Effective number of parameters .....................: 36.93
##
## Marginal log-Likelihood:  -3400.47
## Posterior marginals for linear predictor and fitted values computed
```

```r
summary( result6 )
```

```
##
## Call:
## c("inla(formula = formula5, family = \"binomial\", data = cmvdata, ",  "    control.compute = list(di
##
## Time used:
```

```
##  Pre-processing    Running inla Post-processing           Total
##          0.5723      15944.8615          6.6428      15952.0766
##
## Fixed effects:
##                 mean     sd 0.025quant 0.5quant 0.975quant    mode kld
## (Intercept) -5.8140 0.1974    -6.1993  -5.8161    -5.4175 -5.8199   0
## z_sdi        0.2614 0.0871     0.0922   0.2608     0.4340  0.2596   0
## pct_nw       0.5087 0.3281    -0.1380   0.5094     1.1506  0.5110   0
##
## Random effects:
## Name   Model
##  ID   Besags ICAR model
## zipcode   IID model
## site   IID model
##
## Model hyperparameters:
##                             mean        sd 0.025quant  0.5quant 0.975quant
## Precision for ID      20232.72 22798.650   1587.117 13375.775   80383.75
## Precision for zipcode    14.49    12.270      3.667    10.855      46.91
## Precision for site       10.71     7.747      2.636     8.625      31.12
##                             mode
## Precision for ID      4346.555
## Precision for zipcode    6.987
## Precision for site       5.806
##
## Expected number of effective parameters(std dev): 40.62(18.76)
## Number of equivalent replicates : 2382.94
##
## Deviance Information Criterion (DIC) ...............: 5419.81
## Deviance Information Criterion (DIC, saturated) ....: 5419.78
## Effective number of parameters .....................: 41.81
##
## Marginal log-Likelihood:  -3403.26
## Posterior marginals for linear predictor and fitted values computed
```

Evaluate random effects: (1) Spatially correlated random effects (Zip Code adjacency) (2) Spatially uncorrelated random effects by zip (IID of Zip Code) (3) Spatially uncorrelated random effects by site (IID of Site)

```
temp3a <- data.frame( cbind( "zipcode" = result2$.args$data$zipcode ,
                             "CAR_zip" = result2$summary.random$ID$mean ) )
```

```
## Warning in cbind(zipcode = result2$.args$data$zipcode, CAR_zip =
## result2$summary.random$ID$mean): number of rows of result is not a multiple
## of vector length (arg 2)
```

```
temp3b <- data.frame ( temp3a %>% group_by( zipcode ) %>%
                         summarize( mean = mean( CAR_zip ) ) )
zipcodes$CAR <- temp3b$mean

ggplot( ) +
  geom_sf( data=zipcodes , aes( fill = CAR ), color="NA" ) +
  scale_fill_viridis( option="D" , "Mean"  ) +
  geom_sf( data=st, fill="NA" , color="gray" ) +
  coord_sf( xlim = c( -90000 , 1820000 ) , ylim= c( 880000 , 2150000 ) ) +
  theme_bw( ) +
```

```
  ggtitle( "Spatially Correlated Error" )
```

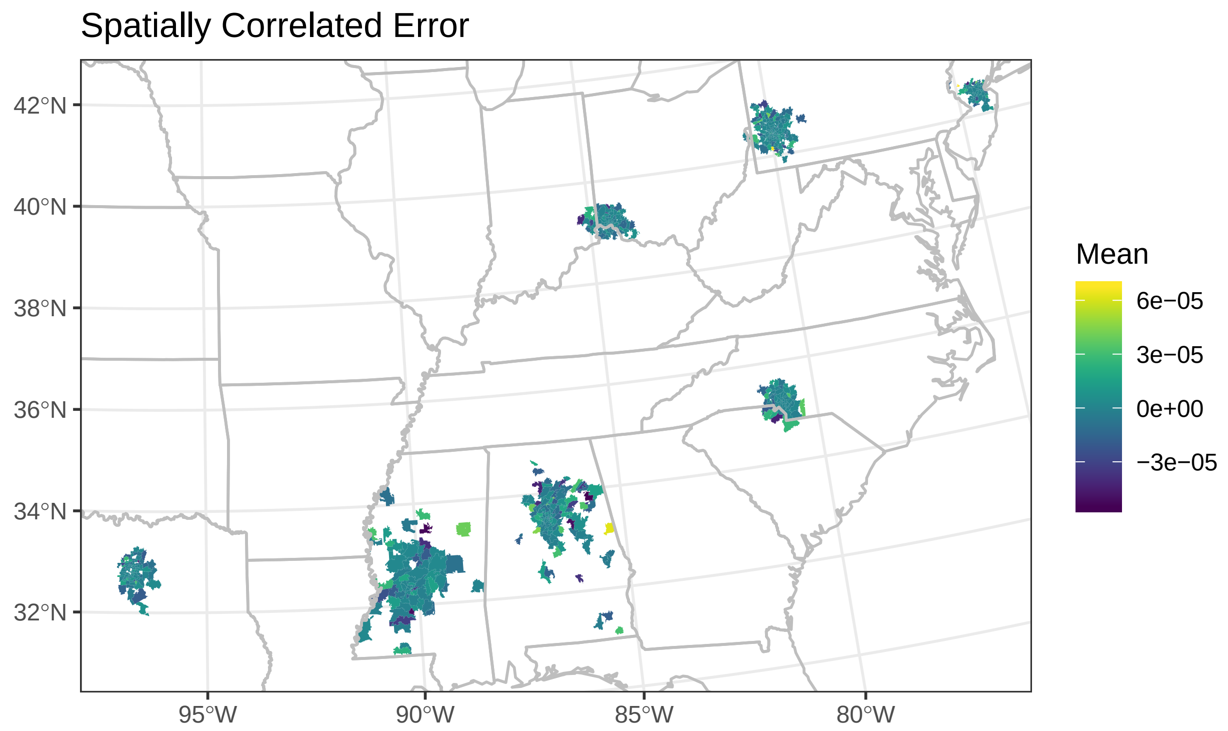


```
temp4a <- data.frame( cbind( "zipcode" = result2$.args$data$zipcode ,
                             "IID_zip" = result2$summary.random$zipcode$mean ) )
```

```
## Warning in cbind(zipcode = result2$.args$data$zipcode, IID_zip =
## result2$summary.random$zipcode$mean): number of rows of result is not a
## multiple of vector length (arg 2)
```

```
temp4b <- data.frame ( temp4a %>% group_by( zipcode )
                       %>% summarize( mean = mean( IID_zip ) ) )
zipcodes$IID_zip <- temp4b$mean

ggplot( ) +
  geom_sf( data=zipcodes , aes( fill= IID_zip ), color="NA" ) +
  scale_fill_viridis( option="D" , "Mean"  ) +
  geom_sf( data=st, fill="NA" , color="gray" ) +
  coord_sf( xlim = c( -90000 , 1820000 ) , ylim= c( 880000 , 2150000 ) ) +
  theme_bw( ) +
  ggtitle( "Spatially Uncorrelated Error" )
```

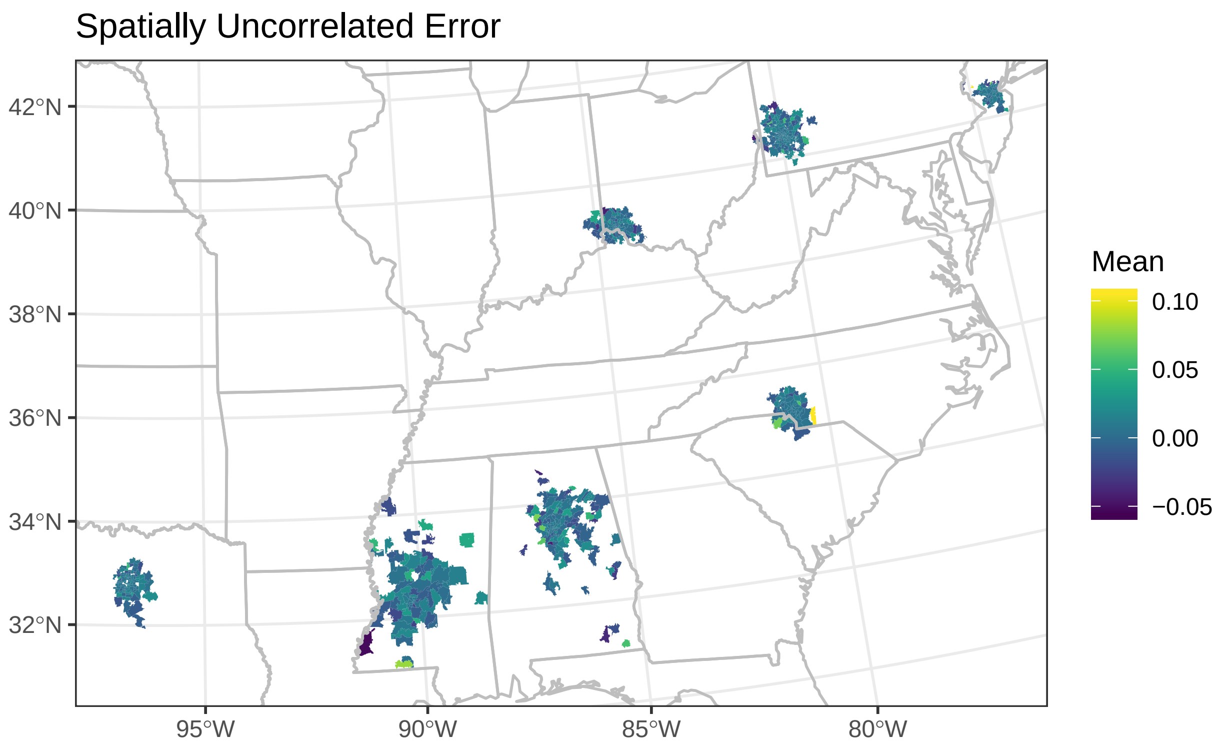


```
site <- cbind( site , "IID"=result1$summary.random$site$mean )

ggplot( ) +
  geom_sf( data=site , aes( fill= IID ), color="NA" ) +
  scale_fill_viridis( option="D" , "Mean"  ) +
  geom_sf( data=st, fill="NA" , color="gray" ) +
  coord_sf( xlim = c( -90000 , 1820000 ) , ylim= c( 880000 , 2150000 ) ) +
  theme_bw( ) +
  ggtitle( "Spatially Uncorrelated Error by Study Site" )
```

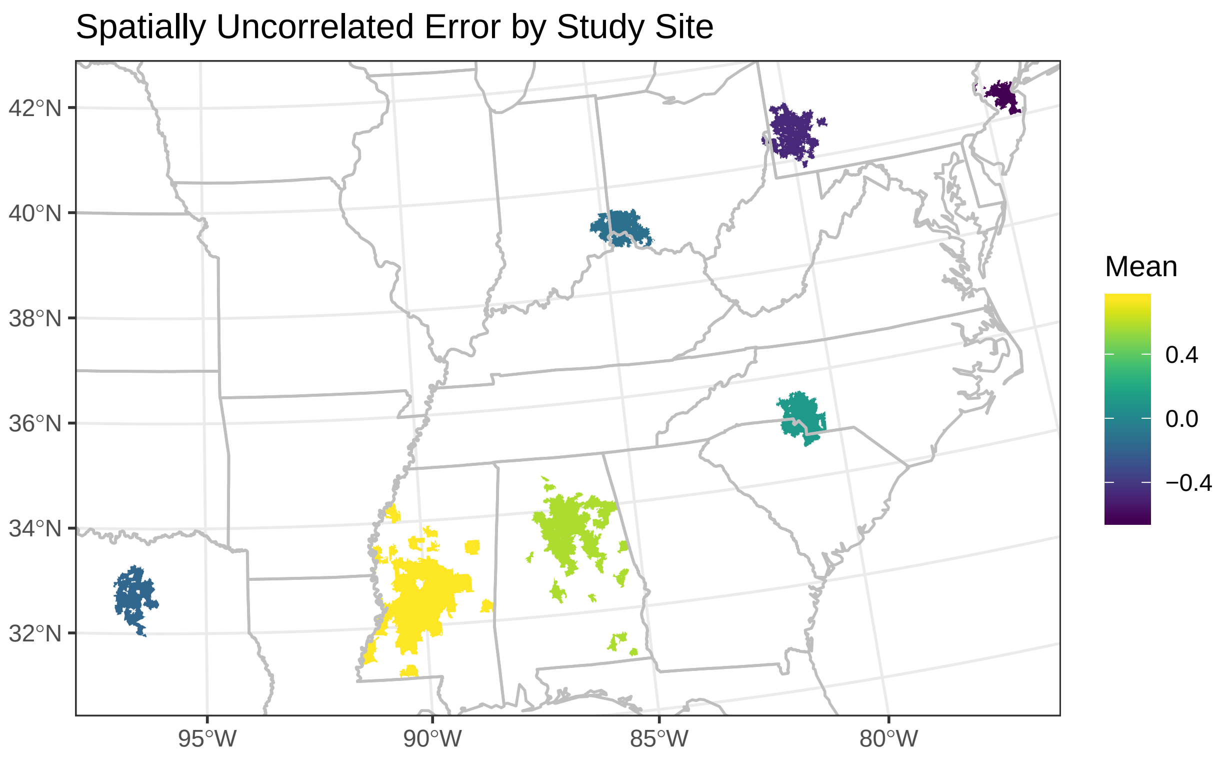

Spatially Uncorrelated Error by Study Site
42°N
40°N
38°N
36°N
34°N
32°N
95°W
90°W
85°W
80°W
Mean
0.4
0.0
−0.4
